# Ponytail Left Anterior Descending Artery: A Case Report

**DOI:** 10.21470/1678-9741-2023-0260

**Published:** 2024-07-15

**Authors:** Rongchuan Yue, Zaiyong Zheng, Zhan Lv, Jie Feng, Houxiang Hu

**Affiliations:** 1 Department of Cardiology, The Affiliated Hospital of North Sichuan Medical College, Nanchong, Sichuan, China.; 2 Nucleic Acid Medicine of Luzhou Key Laboratory, Luzhou, Sichuan, China.; 3 Department of Cardiology, The Affiliated Hospital of Southwest Medical University, Luzhou, Sichuan, China.

**Keywords:** Coronary Vessel Anomalies, Pathologic Construction, Coronary Angiography, Computed Tomography Angiography

## Abstract

Division of the anterior descending branch into many small arteries is a rare coronary anomaly. We report the case of a 64-year-old female with severe stenosis (>75%) in the proximal region of the anterior descending branch as indicated by coronary computed tomography angiography (CCTA). In addition, coronary angiography showed that the anterior descending branch of the coronary artery split into numerous small arteries, an anomaly that can confound clinical examination.

## INTRODUCTION

Cardiovascular disease is the leading cause of mortality and morbidity worldwide. Given that coronary blood flow can show significant changes prior to cardiac dysfunction and/or structural disorders, it is crucial to evaluate coronary patency using coronary computed tomography angiography (CCTA) and coronary angiography via percutaneous coronary intervention (PCI). With the widely application of PCI and CCTA, more and more artery anomalies have been reported. Herein, we present a previously undescribed coronary artery anomaly in which the left anterior descending (LAD) branch is divided into many small arteries, resembling a ponytail. This unique 'ponytail' coronary anomaly may pose challenges for radiological interpretation and clinical examination. We report the case of a 64-year-old female with severe stenosis (>75%) at the proximal region of the anterior descending branch revealed by CCTA. In addition, coronary angiography revealed that the anterior descending branch was divided into numerous small arteries.

## CASE PRESENTATION

**Fig. S1:**
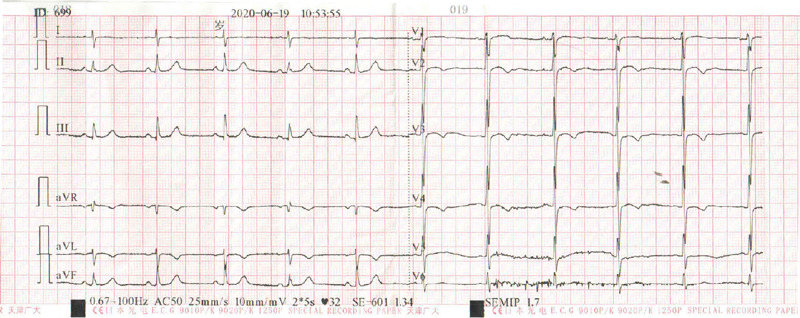
The twelve-lead ECG on admission showed T-wave inversion in leads V3-5.

**Fig. 1:**
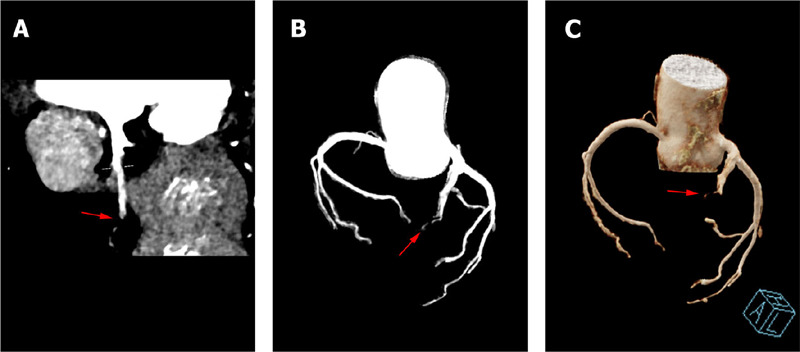
CCTA imaging (A-B) and three-dimensional reconstruction (C) revealed severe stenosis (>75%) at the proximal region of the anterior descending branch.

A 64-year-old female was admitted to our hospital wih an ischial tuberosity cyst. Twelve-lead electrocardiograms (ECGs) revealed T-wave inversion in V3-5 (Figure S1). CCTA was subsequently performed, which indicated proximal stenosis (>75%) of the anterior descending branch (Figure 1). 

**Fig. 2:**
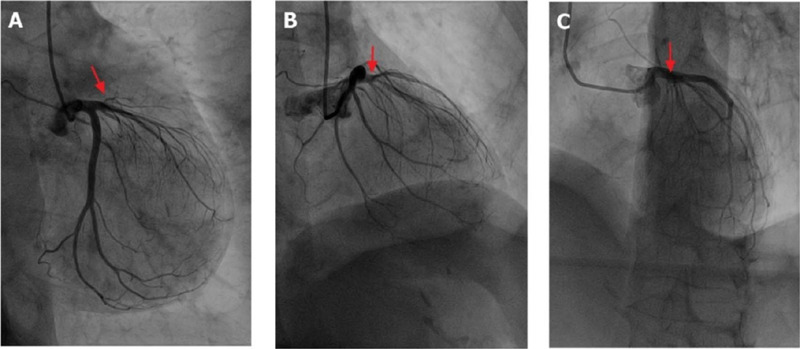
A-C show three sample images captured with the C-arm oriented at the anterior descending branch view at 30 degrees (RAO30) and CAU30. The images show the anterior descending branch divided into several small arteries.

Physical examination and laboratory screening results were normal, and echocardiography revealed no abnormalities. The patient was transferred to the cardiology ward for coronary angiography, which revealed that the anterior descending branch was divided into many small arteries like a ponytail (Figure 2 and videos 1 to 5). The left circumflex and right coronary arteries were normal. The anomaly appeared benign, and the patient was able to undergo cystectomy. After 12 weeks of follow-up, the patient remained healthy without any symptoms.

**Video 1-4:**
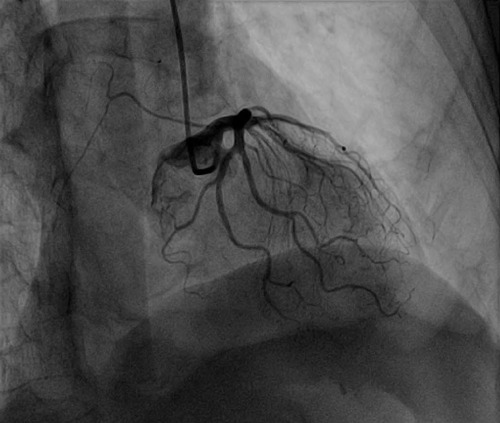
Images were captured with the C-arm oriented at the anterior descending branch at “ponytail” anterior descending branch view with different angles.

*Link 1: https://s3.sa-east-1.amazonaws.com/publisher.gn1.com.br/bjcvs.org/videos/Video-1.avi*

*Link 2: https://s3.sa-east-1.amazonaws.com/publisher.gn1.com.br/bjcvs.org/videos/Video-2.avi*

*Link 3: https://s3.sa-east-1.amazonaws.com/publisher.gn1.com.br/bjcvs.org/videos/Video-3.avi*

*Link 4: https://s3.sa-east-1.amazonaws.com/publisher.gn1.com.br/bjcvs.org/videos/Video-4.avi*

**Video 5:**
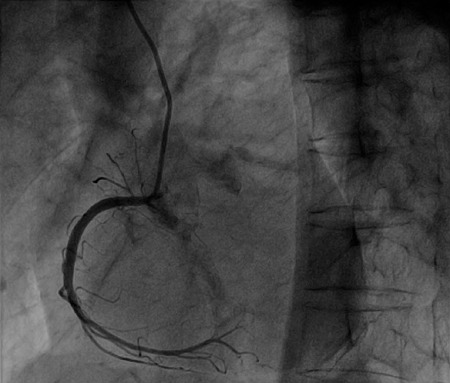
Images were captured with the C-arm oriented at the right coronary artery view, LAO45°.

*Link: https://s3.sa-east-1.amazonaws.com/publisher.gn1.com.br/bjcvs.org/videos/Video-5.avi*

## DISCUSSION

Coronary artery anomalies (CAAs) are rare, with a prevalence of 0.64-1.3% in coronary angiographies. With the advent of CCTA, more CAAs have been detected, with incidence rates ranging between 0.7% and 18.4%. To distinguish non-pathogenic variants from potentially disease-causing variants, Angelini et al.^[1]^ proposed defining the “normal” coronary artery as those present in >1% of an unselected general population, which contains normal coronary arteries and normal anatomical variants^[2]^. 

**Fig. 3:**
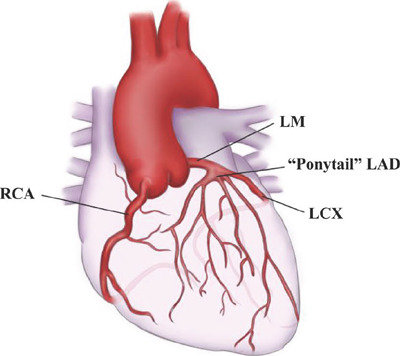
The anterior descending branch is divided into many small arteries from the left main coronary. RCA=Right coronary artery; LM=Left main coronary; LCX=Left circumflex branch; LAD=Left anterior descending

Dual left anterior descending (LAD) is one of the most common anomaies in LAD, although current diagnosis and classification strategies limit the number of LAD branches^[3]^. Unlike duplicated LAD, in this patient, the anterior descending branch was divided into a large number (≥3) of small arteries (Figure 3), all originating from the left main stem, like a "ponytail”. This abnormality has never been documented before and does not fit within the traditional classification system.

Most CAAs are incidentally detected during radiographic examinations and may partly resemble thrombotic recanalization on angiographic examination^[4]^ or develop after severe stenosis^[5,6]^. However, the patient did not report prior history of myocardial infarction, and echocardiography did not reveal any structural or functional abnormalies. Furthermore, the position and direction of the blood vessel suggested anatomical variation rather than thrombotic recanalization.

CAAs are the second most common cause of sudden cardiac death in young athletes^[2]^. In the present case, the small branches provided adequate hemodynamic support in the patient’s daily life. However, ponytail LAD can be fatal in the setting of atherosclerosis. Furthermore, such abnormalities may lead to erroneous clinical diagnoses. In our case, the anterior descending branch was divided into many small branches, and CCTA revealed severe stenosis at the proximal region of the anterior descending branch.

With several CAAs, the risks and benefits of therapy, especially surgery, should be considered carefully^[7]^. Although non-invasive imaging can detect multiple CAAs, not every anomaly affects patient outcomes, and inappropriate treatment procedures may have adverse effects^[8]^. Additionally, multiple imaging methods, such as intravascular ultrasound (IVUS), should be used for more detailed diagnoses. IVUS can provide accurate information about the anomalous vascular structure, distinguishing coronary anomalies from thrombus recanalization. Unfortunately, our patient refused to participate in further investigation.

## CONCLUSION

We report a previously unknown anomaly of the coronary artery wherein the anterior descending branch is divided into several small arteries. 

### Ethics Approval and Participant Consent

The experimental protocol followed the guidelines of the Declaration of Helsinki. Waivers of Informed Consent were approved by the Human Ethics Committee of the Affiliated Hospital of North Sichuan Medical College. 

## Abbreviations, Acronyms & Symbols

CAAs: Coronary artery anomalies
CTA: Computed tomography angiography
CCTA: Coronary computed tomography angiography
ECG: Electrocardiogram
ESC: European Society of Cardiology
IVUS: Intravascular ultrasound
LAD: Left anterior descending
MPI: Myocardial perfusion imaging
MRI: Magnetic resonance imaging
PCI: Percutaneous coronary intervention
